# Supplementary material for: A Genome‐Wide Association Study of Non‐Photochemical Quenching in response to local seasonal climates in Arabidopsis thaliana
Source: Plant Direct. 2019 May 23;3(5):e00138. doi: 10.1002/pld3.138 (PMC6603398; doi:10.1002/pld3.138)
Supplement: Supplementary file 1 [file PLD3-3-e00138-s001.pdf]

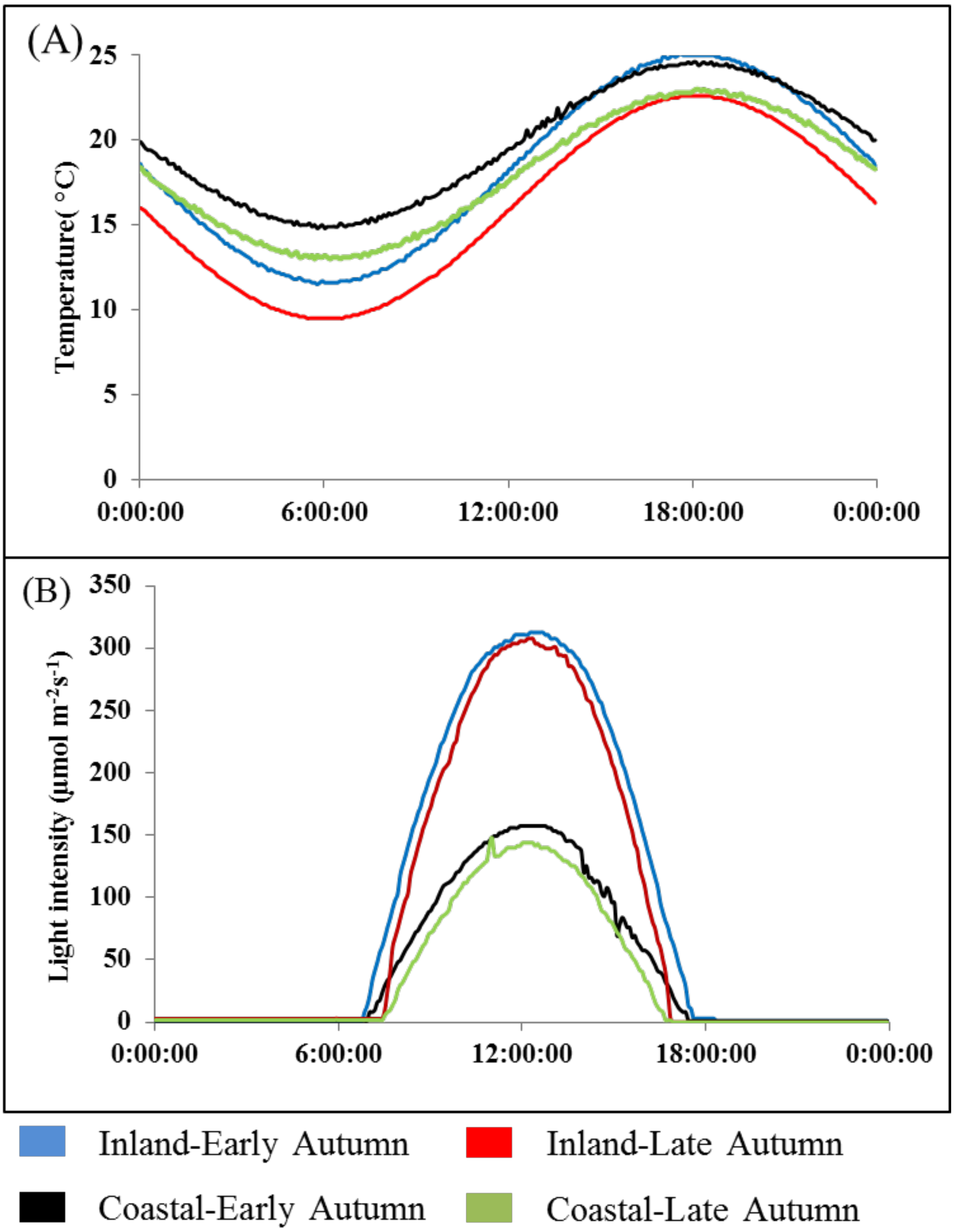

S1. Model of simulated Coastal and Inland conditions in early- and late-autumn showing dynamic changes in temperature (A) and light intensity (B

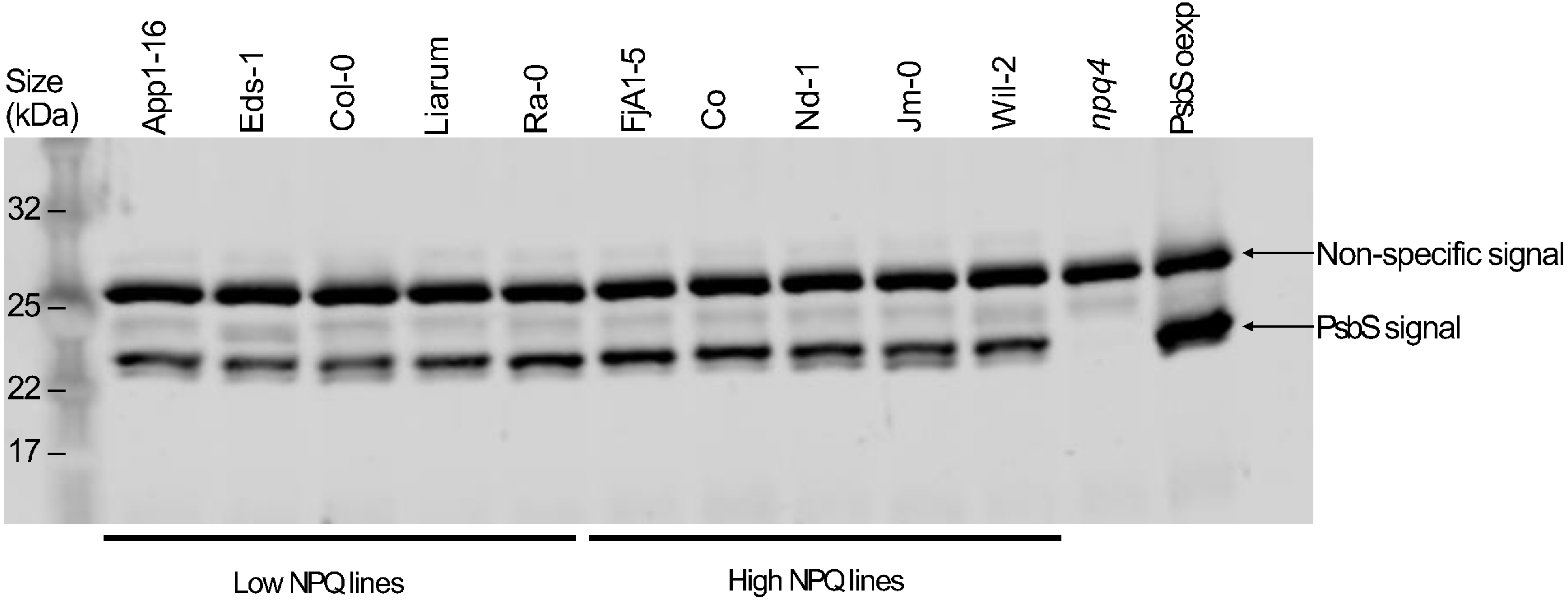

S2. Sample western blot showing the PsbS protein content of five low- and high-NPQ plant lines, as well as the *npq4* and PsbS overexpression mutants. The non-specific signal was consistent across all measurements and used to quantify the amount of PsbS protein present in the plant leaf tissue

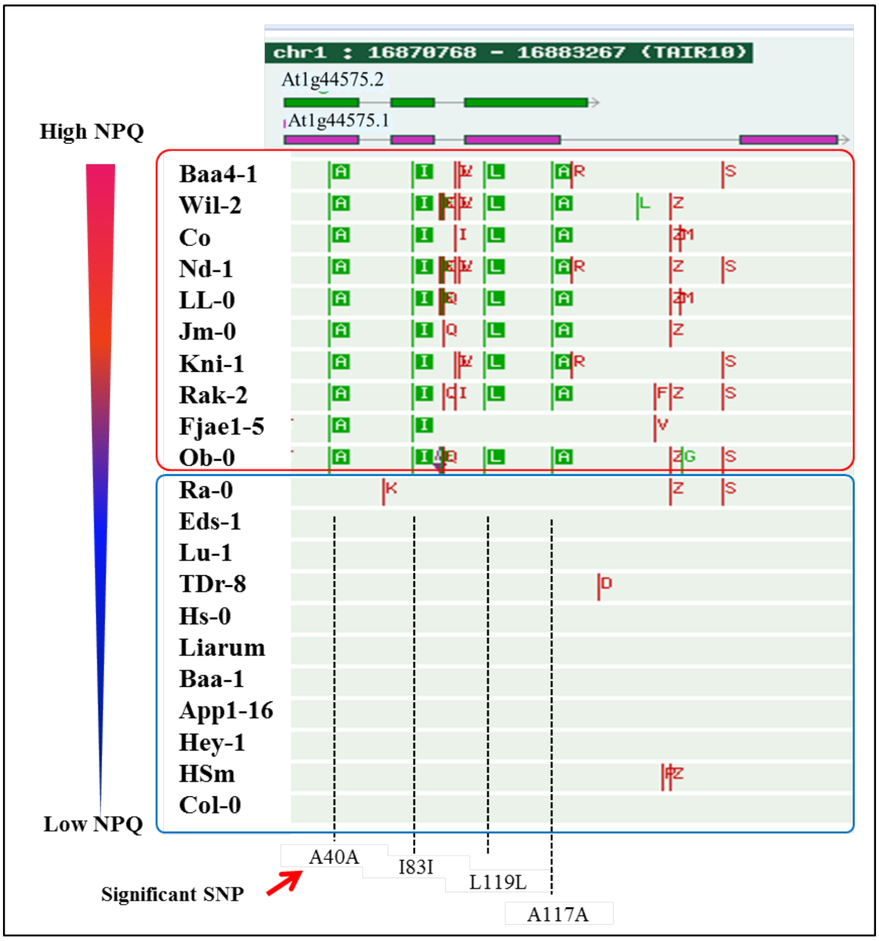

S4. Haplotype mapping of high and low NPQ accessions showing amino acid change at the SNPs peaks: synonymous amino acids showed as green colour. Data was generated using online software: *http://signal.salk.edu/atg1001/3.0/gebrowser.php*.

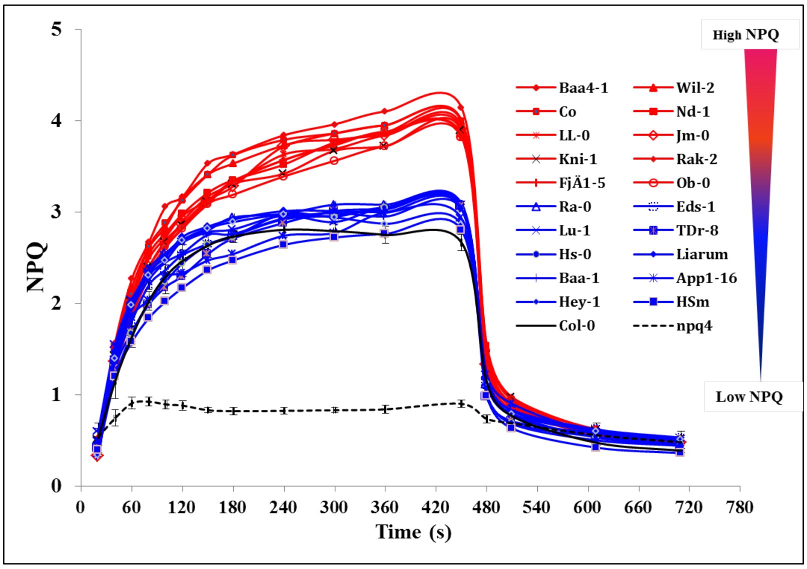

S6. NPQ induction of high (red) and low (blue) NPQ haplotypes under Late Autumn Coastal conditions at the 16 leaves stage. The mean + S.D. is given for Col-0 (n=16) and *npq4* (n=4) with the other genotypes represented by one plant.

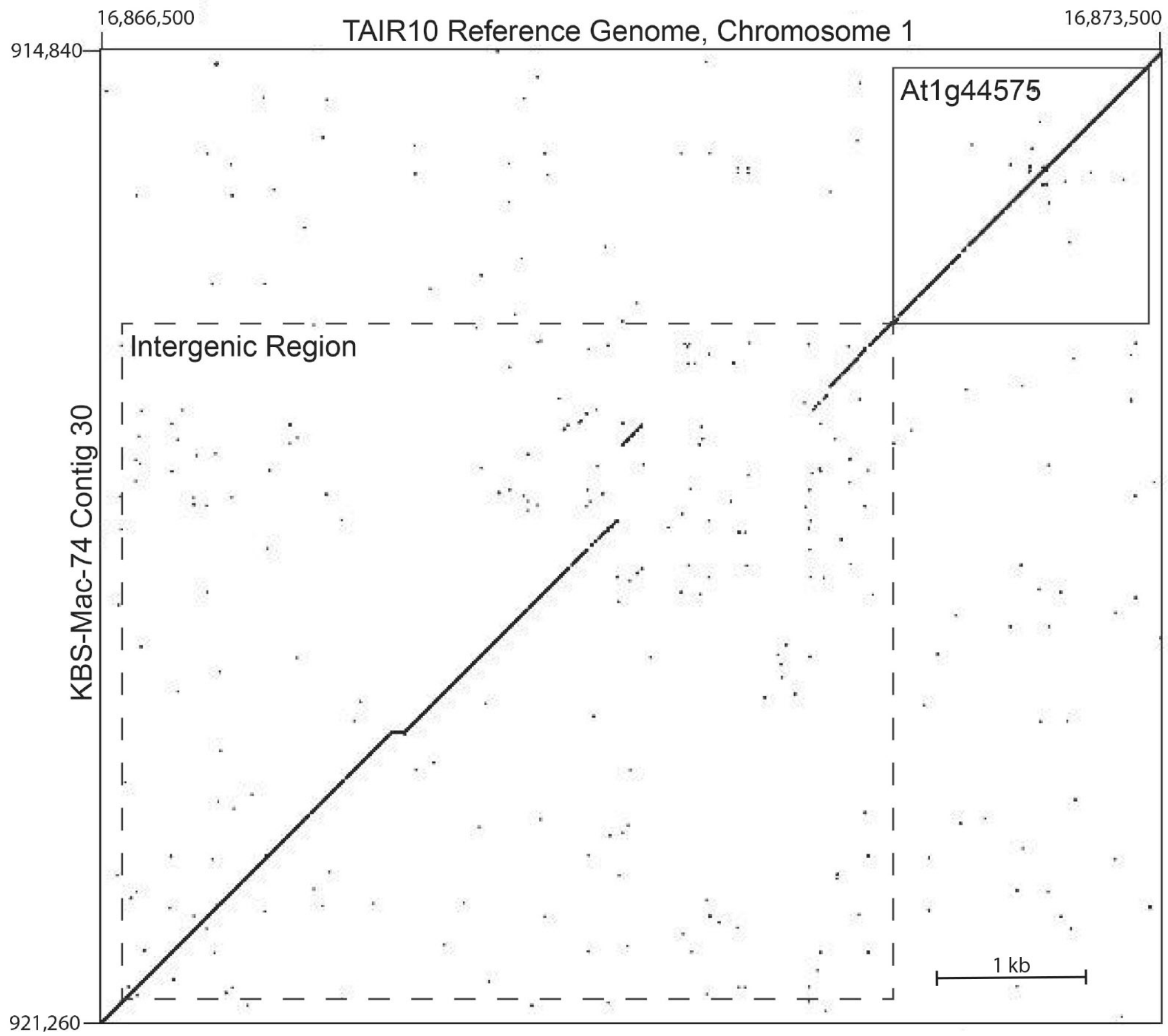

S7. Dot matrix comparing *PsbS* genomic regions of TAIR 10 Col-0 Reference Genome and KBS-Mac-74. Col-0 is representative of low NPQ accessions and KBS-Mac-74 is representative of a high NPQ accessions. The *PsbS* gene (At1g44575) and the intergenic regions of the respective genome alignment are labelled with solid-line and dashed-line boxes, respectively. The obvious discontinuity in the intergenic region may indicate the causative agent for the NPQ differences observed in this study.
