# Supplementary material for: A Genome‐Wide Association Study of Non‐Photochemical Quenching in response to local seasonal climates in Arabidopsis thaliana
Source: Plant Direct. 2019 May 23;3(5):e00138. doi: 10.1002/pld3.138 (PMC6603398; doi:10.1002/pld3.138)
Supplement: Supplementary file 3 [file PLD3-3-e00138-s003.pdf]

comments inline below.
Reviewer #1:
Simple additional ref
**The manuscript has improved substantially since review, and I think the conclusions and limits of the study are better defined. One last comment: it looks like a part of this dataset was already presented in a previous publication, (Fig. 5E, Rungrat et al. 2016), albeit with a different analysis. That should be mentioned in the methods.**

This is currently referenced in the methods on line 153-154.

Rungrat T, Awlia M, Brown T, et al. Using Phenomic Analysis of Photosynthetic Function for Abiotic Stress Response Gene Discovery. Arabidopsis Book. 2016;14:e0185. Published 2016 Sep 9. doi:10.1199/tab.0185

Reviewer #2:

**Studies with plants are favored by the possibility of designing experiments with replications of the "same individual" (hybrids/homozygote lines/clones), which is not possible with humans (although, most human studies are case-control studies, with individuals in each group being used as replication of a given condition such as healthy or unhealthy individuals). Even with populations of unique individuals such as segregating populations (e.g. F2), one might explore unreplicated designs such as augmented block design or partially replicated designs. In such cases, means of unreplicated treatments are adjusted based on the replicated ones that are used to estimate residual variance. The authors say that the replicates of Col-0 were included to estimate variation. If raw phenotypes are being used in the model, how is this information being accounted for? This should be made clear.**

Our experimental design aimed to maximize genotypic variation across distantly related accessions so SNPs could be evaluated for association across distinct backgrounds. This panel was used across multiple environments. A reference accession Col-0 was included multiple times in each environment to estimate within and between environmental variation as described. The reference accession values were not used to adjust raw values of other accessions as this would not change the analysis within each environment. For the GxE analysis, our prior experience is that correction of a large population ~300 using a few reference replicates 3-6 within each environment made no difference or worse introduced noise. We feel that describing this alternative detail would introduce confusion.

**Line 222: The correct expression for Bonferroni correction should be alpha/m, where m is the number of markers in this case. But since a permutation test was performed, I suggest using permutation instead of Bonferroni correction, which should be less conservative.**

We have corrected the formula for Bonferroni. Line 222. Because the SNPs have been pruned to an independent set., the permutation threshold was very similar to Bonferroni.

**In many cases, the K model alone does not efficiently account for population structure. In such case, a Q+K model would be required. Please include the qqplots as supplementary files to show the adequacy of the model being used.**

We have only used the K model to account for population structure. This has been determined to most appropriate and sufficient for Arabidopsis studies using this balanced population [Atwell, 2010, Li et al, 2010, 2014, Baxter et al 2010] QQ plots were generated and were similar to previous analysis (attached).

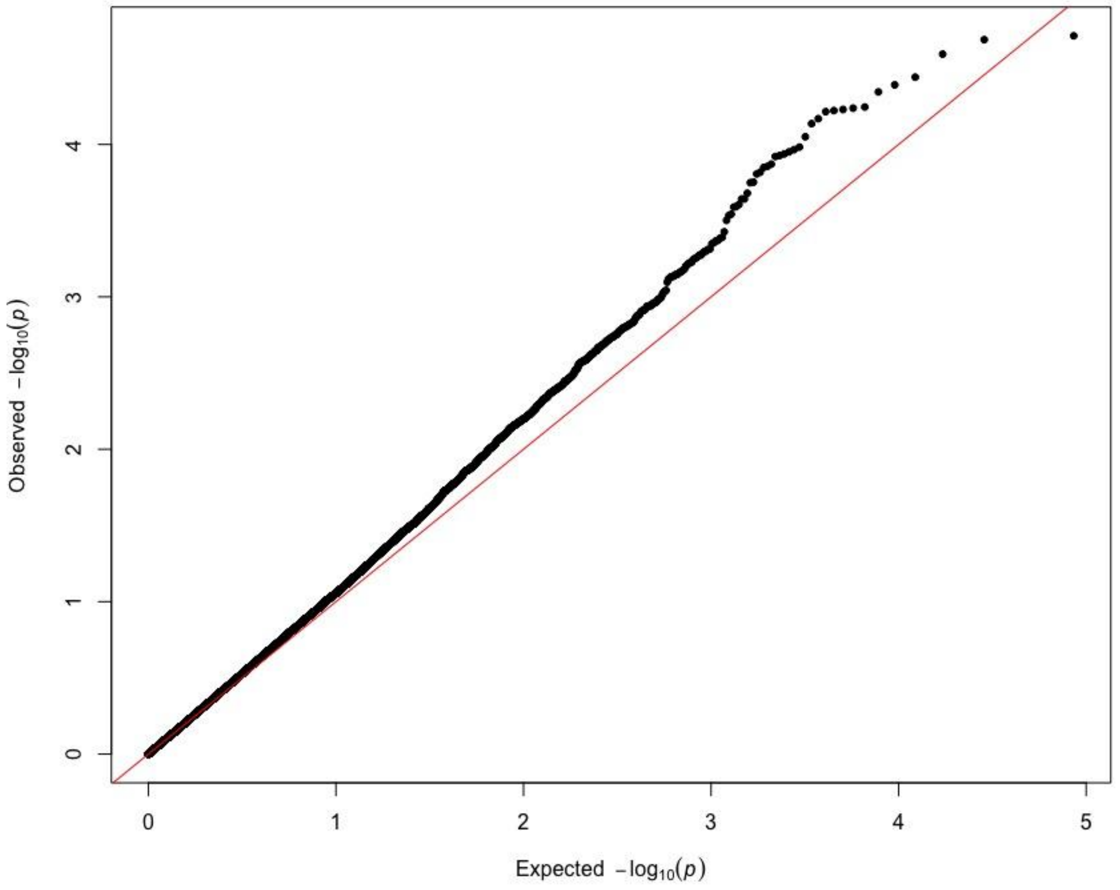


**The threshold of 2.5% might be low, it means ~8 alleles present in the dataset would be enough to pass the filter. I suggest the authors either use a more stringent filter (such as the more commonly used 5%) or show the minor allele frequency of top SNPs. This would avoid the possibility of having false positives caused rare alleles.**

Thank you for this suggestion, but we prefer to stick with our current threshold which we feel from prior experience this balances the false positive and false negative rates (missing rare alleles of large effect)
